# Application of a Fluctuating Charge Polarization Model to Large Polyaromatic Hydrocarbons and Graphene Nanoflakes

**DOI:** 10.1021/acs.jpclett.3c02013

**Published:** 2023-08-28

**Authors:** Devin M. Mulvey, Kenneth D. Jordan

**Affiliations:** Department of Chemistry, University of Pittsburgh, 219 Parkman Avenue, Pittsburgh, Pennsylvania 15260, United States

## Abstract

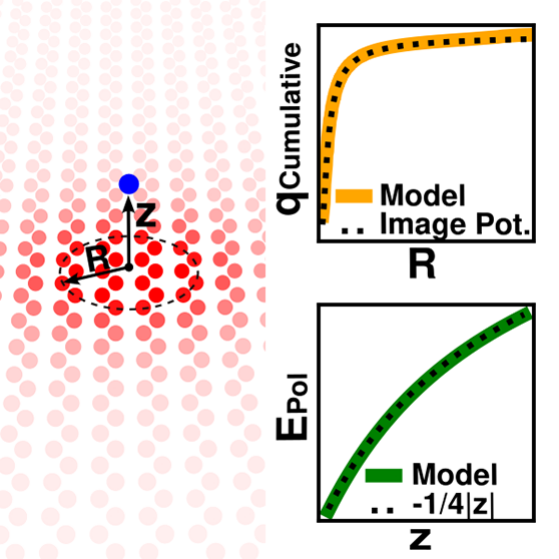

We present a polarization model incorporating coupled fluctuating charges and point inducible dipoles that is able to accurately describe the dipole polarizabilities of small hydrocarbons and, for sufficiently large graphene nanoflakes, reproduce the classical image potential of an infinite conducting sheet. When our fluctuating charge model is applied to the hexagonal carbon nanoflake C_60000_ we attain excellent agreement with the image potential and induced charge distribution of a conducting sheet. With the inclusion of inducible dipole terms, the model predicts an image plane of *z*_im_ = 1.3334 a_0_, which falls in line with prior estimates for graphene. We consider the case of two charges placed on opposite sides of C_60000_ and find that the fluctuating charge model reproduces classical electrostatics once again. By testing opposing and similar signs of the external charges, we conclude that an atomically thin molecule or extended system does not fully screen their interaction.

The explicit inclusion of polarization in force fields is essential for an accurate description of intermolecular interactions and adsorption of atoms and molecules at interfaces, particularly when the species interacting with the interface are charged or highly polar. Inclusion of polarization is also essential in model Hamiltonian approaches to nonvalence anions of molecules and molecular clusters.^1−5^ Most polarizable force fields treat only local polarization, most commonly through point inducible dipoles^6−13^ or Drude oscillators (sometimes referred to charge-on-spring models).^7,14−16^ Such force fields neglect long-range charge delocalization, which becomes increasingly important for molecules with shrinking HOMO–LUMO gaps.

One of the most common approaches to treating charge delocalization is through fluctuating charge (FQ) models^17,18^ based in the concepts of electronegativity equalization^19^ and chemical potential equalization.^20,21^ There are a variety of implementations addressing the shortcomings of the original FQ models,^22−28^ which will be discussed later on. There are models that combine both the local polarization of inducible dipoles with the long-range charge delocalization of FQ,^29−32^ and we have recently shown^33^ that a modified version of the inducible charge-dipole model of Mayer and Åstrand (MÅ),^31,32^ hereafter referred to as MMÅ1, closely reproduces the PBE0^34−37^ and MP2^38^ values of the dipole polarizabilities of hexagonal polyaromatic hydrocarbons (PAHs) belonging to the series . The largest species that we were able to treat with density fitted (DF) PBE0 and MP2 methods were C_294_H_42_ and C_96_H_24_ respectively in ref (33), in which the def2-TZVPD basis set,^39,40^ its Coulomb-exchange (JK),^41^ and resolution of identity (RI)^42^ fitting basis sets were employed. By adopting a trimmed version of the def2-SVPD basis set,^39,40^ along with its JK^41^ and RI^42^ fitting basis sets, we have been able to extend the data set to C_384_H_48_ and C_150_H_30_, at the density fitted PBE0 and MP2 levels, respectively. The trimmed def2-SVPD basis set removes the tight d function from the basis for C and the p functions from the basis for H. A finite field perturbative approach was employed in the program Psi4 v1.4a2^43,44^ to obtain the polarizabilities of the PAHs, the details of which are described in the repository containing all data reported in this study. The polarizability values calculated with the trimmed def2-SVPD basis set are slightly smaller (≤6%) than those calculated with the larger basis set used in ref (33). We report the values of the in-plane polarizability obtained with this smaller basis set along with the results of the MMÅ1 model in Figure 1.

**Figure 1:**
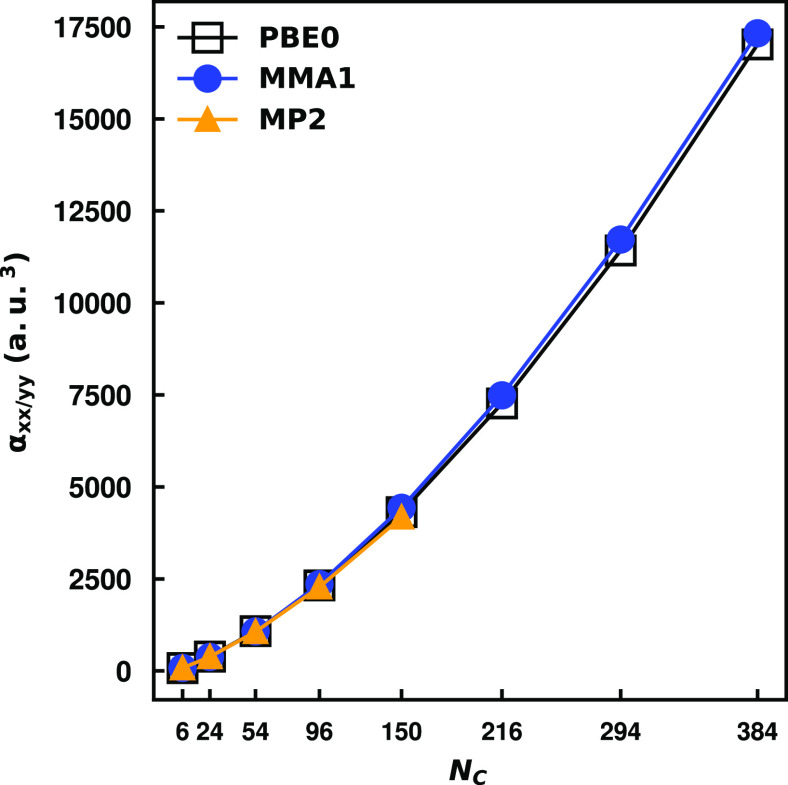
In-plane polarizabilites of hexagonal PAHs up to C_384_H_48_. Results are reported for the PBE0 and MP2 methods using a trimmed version of the def2-SVPD basis set as well as for the MMÅ1 model. The *x*-axis label *N*_*C*_ denotes the number of carbon atoms in the PAH.

As seen from Figure 1, the MP2, PBE0, and MMÅ1 values of the polarizability are in close agreement (within 3%). Reference (33) also reported polarizabilities obtained using the point inducible dipoles only and charge flow terms only in the MMÅ1 model. Those calculations revealed that the contribution from the point inducible dipoles to the in-plane component of the polarizability grows essentially linearly with molecule size, while that due to the charge flow terms grows approximately quadratically with system size. The net values of the in-plane polarizabilities are smaller than the sum of the induced dipole and charge flow terms calculated separately due to the cross term between these two contributions. Although not reported in Figure 1, we note that the MP2, PBE0, and MMÅ1 models all give similar values of the out-of-plane polarizability and that this component grows approximately linearly with system size.

The success of a FQ model in describing the polarizabilities of the hexagonal PAHs is surprising, as it is well-known that such models tend to overestimate long-range charge transfer.^22−27^ This raises the following questions: Is the overestimation of long-range charge transfer more problematic in larger PAHs? Or is the “issue” of overpolarization in FQ models actually moot due to the shrinking HOMO/LUMO gap and concomitant increase in metallic character with increasing size of the PAHs? Indeed, the documented problems of FQ models, overpolarization and exaggerated charge flow between atoms separated by large distances, arise when applied to molecular systems with insulating behavior.^22,23,27^ In this work, we address these questions by examining how closely the polarization potential between a point charge (*Q*) and large hexagonal carbon nanoflakes belonging to the series  calculated with the MMÅ1 model approaches the classical image potential, −*Q*^2^/(4|*z*|) (in atomic units), where the molecule is taken to be in the *xy* plane. The absolute value of *z* is used because the systems considered are isolated from a substrate and are symmetric with respect to reflection through the *xy* plane. We also compare the induced charge distributions obtained from our model with those for an infinite conducting sheet.

The polarization energy (*E*_pol_) expression in the MÅ model is given by
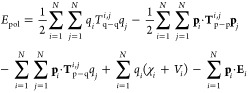
1where *q*_*i*_ refers to the induced charge, **p**_*i*_ the induced dipole, and χ_*i*_ the atomic electronegativity of atom *i*. The quantities *V*_*i*_ and **E**_*i*_ represent, respectively, the values of an external potential and electric field at atoms *i* and *N* as the number of atomic sites. In the present study, we are not concerned with permanent charges on atoms, so terms coupling atomic electronegativities χ_*i*_ to induced charges (fourth sum in eq 1) do not contribute to the polarization energy. The *T*^*i*,*j*^ and **T**^*i*,*j*^ factors represent dampened electrostatic interaction functions between the induced multipoles. These functions are derived with the assumption that the atomic charge distributions are well described by Gaussian functions. Of special interest are the *T*_q–q_^*i*,*j*^ factors, defined as
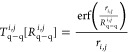
2where *r*_*i*,*j*_ is an interatomic distance and *R*_q−q_^*i,j*^ = . The free parameters, *R*_q_^*i*^ and *R*_q_^*j*^, are the radii of the charge distributions of atoms *i* and *j*, respectively. When *i* = *j* and *r*_*i*,*j*_ → 0, *T*_q–q_^*i*,*i*^ determines the atomic hardness, which is associated with the self-energy required to assemble the isolated atomic charge distribution. Likewise, the self-energy required to assemble an isolated atomic induced dipole is obtained from the terms involving **T**_p–p_^*i*,*j*^ when *r*_*i*,*j*_ → 0. The charge–charge (*T*_q–q_^*i*,*j*^), charge–dipole/dipole–charge (*T*_p–q_^*i*,*j*^), and dipole–dipole (*T*_p–p_^*i*,*j*^) expressions for damping interactions between induced moments on separate atoms and the corresponding self-energy expressions when *r*_*i*,*j*_ → 0 are explained in detail in the works of Mayer and Åstrand.^31,32^

The primary difference between the original MÅ and MMÅ1 models is in the choice of *R*_q_ for a carbon atom, which was increased from 0.0303 a_0_ (MÅ) to 0.2365 a_0_ (MMÅ1). This change was essential for obtaining dipole polarizabilities in good agreement with the PBE0 and MP2 results for the hexagonal PAHs considered in ref (33). It is important to note that for both the MÅ and MMÅ1 models the resulting *R*_q–q_^*i*,*j*^ values provide essentially no attenuation to the Coulomb interaction of charges between different atomic sites, even in the case of directly bonded carbon atoms. Other differences between the original MÅ model and the MMÅ1 model involve small adjustments in the atomic dipole polarizabilities and a modification of the damping of the charge–dipole and dipole–dipole interaction terms. The details are given in ref (33).

Figure 2a,b reports the polarization potentials calculated using the MMÅ1 model, retaining the charge flow terms only, for a unit point charge located from 5 to 10 a_0_ above the plane of atoms along the principal rotational axis of hexagonal carbon nanoflakes: C_150_, C_600_, C_2400_, C_15000_, and C_60000_. Obviously, the electronic properties of graphene nanoflakes depend on the edge termination. In the context of the MMÅ1 model, the polarization potentials along the principal rotational axes are little affected by the removal of the H atoms, so these calculations can be viewed as being appropriate for hexagonal PAHs, , which can be viewed as H-terminated graphene nanoflakes. The figure also includes the −1/(4|*z*|) potential from classical image potential theory (note that the figures report the potentials in eV).

**Figure 2:**
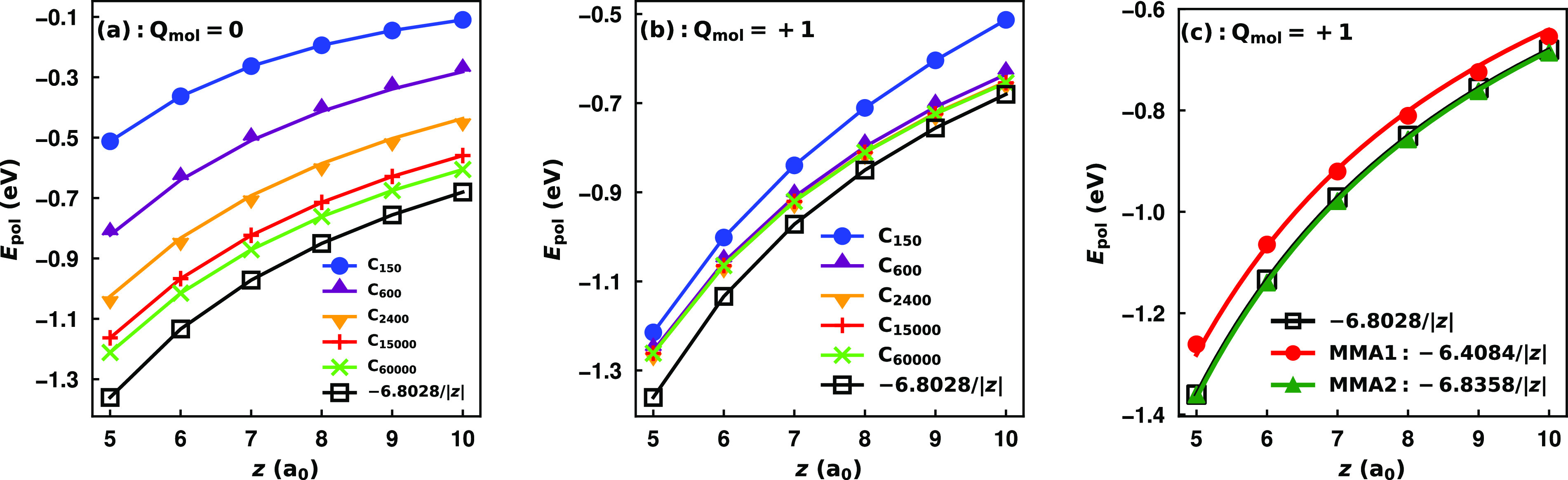
Polarization potential of a negative point charge (*Q* = −1) interacting with increasingly large hexagonal carbon nanoflakes calculated using the charge flow term of the MMÅ1 and MMÅ2 models. Results show the cases where the nanoflakes are constrained to have (a) a total charge of *Q*_mol_ = 0 and (b) a total charge of *Q*_mol_ = 1 using the MMÅ1 model. In (c) the MMÅ1 and MMÅ2 models are compared using a grounded (*Q*_mol_ = 1) C_60000_ nanoflake. In all images, comparison is made with the classical image potential for a conducting sheet, −1/(4|*z*|) in a.u.

The classical image potential is derived for a grounded sheet, which means that a negative unit charge above the surface induces a +1 charge on the surface. However, the same potential is expected for the ungrounded sheet, since the induced charge can be “harvested” from infinite distances. In reporting results for the MMÅ1 model, we considered both the grounded (net charge = 1) and ungrounded (net charge = 0) limits. We recognize that an induced charge equal in magnitude, but opposite in sign, from the interacting point charge for a finite “grounded” graphene nanoparticle or PAH is unphysical for a nonzero HOMO/LUMO gap, but should be physical in the limit of a zero gap. We also note that comparison with the classical image potential only makes sense for z values much less that the radius of the finite system considered.^45^ By limiting z values to 5 to 10 a_0_, this condition is satisfied for all of the systems reported in the figure except C_150_.

As seen from Figure 2a, for the ungrounded nanoflake, convergence of the polarization potential with increasing system size is very slow, with significant differences between the potentials for C_15000_ and C_60000_. The polarization potential from the charge flow portion of the MMÅ1 model for C_60000_ is less attractive than −1/(4|*z*|) with the difference being as much as 11% over the range of separations considered. The results for the nanoflakes constrained to have a charge of +1 are shown in Figure 2b, from which it is seen that the convergence of the potential calculated using the MMÅ1 model with system size is much more rapid for the grounded case than for the ungrounded case. Indeed, when *Q*_mol_ is constrained to be +1, the potential for C_600_ is close to that of C_60000_. However, even for C_60000_ the potential from the MMÅ1 model is up to 7.3% smaller in magnitude than the image potential result of −1/(4|*z*|). This suggests a limitation in the parametrization of the MMÅ1 model.

In considering factors that could cause the discrepancy between the polarization potential of C_60000_, as described by the MMÅ1 model, from the −1/(4|*z*|) image potential result, it occurred to us that this may be a consequence of the model having essentially no damping of charge–charge interactions. To explore this possibility, we multiplied the *T*_q–q_^*i*,*j*^ (where *i* ≠ *j*) terms by a factor of , while keeping all other parameters of the MMÅ1 model fixed.

As seen in Figure 2c, with the inclusion of the damping factor, with α = 0.4191, the charge flow term in the modified MÅ model gives a polarization potential for C_60000_ that is essentially identical to that from the classical image potential when the nanoflake is assumed to be grounded. Hereafter, we refer to the model with additional charge–charge damping as MMÅ2. With the net charge of the nanoflake constrained to be zero, the MMÅ2 model gives a polarization potential for C_60000_ only about 4% less attractive than the image potential result. Presumably, this reflects the need to use an even larger nanoflake to converge the potential from the MMÅ2 model to the image potential result when the nanoflake is constrained to be charge neutral. The inclusion of the damping factor leads to a moderate (≤9%) increase of the in-plane polarizabilities of the hexagonal PAHs up to C_384_H_48_ in size compared to the PBE0 and MP2 results. While this could be remedied by making *R*_q_ size dependent, changing with the HOMO–LUMO gap of the system, we have chosen instead to leave *R*_q_ unchanged.

Another way of assessing the performance of the MMÅ2 model for describing the polarization of large hexagonal nanoflakes is to examine the charge distributions induced by a point charge above the plane of the molecule. In this context, we compare the charge distributions induced by a point charge interacting with the C_60000_ nanoflake, as described by the charge flow contribution of the MMÅ2 model and a grounded, conducting slab. In the case of the nanoflake, the point charge is located on the main rotational axis, while for the slab it is located at (0, 0, *z*). The expression for the induced charge density at the surface of a grounded slab induced by a point charge *Q* at (0, 0, *z*) is given by
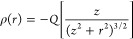
3where *r* is the radial distance in the plane of the surface measured from the origin  and *Q* is the value of the external charge. This expression makes use of polar coordinates and includes the integration over the angle ϕ from 0 to 2π radians. One can obtain the cumulative charge enclosed within a finite radius *R* by integrating eq 3 from the origin up to *R*, which gives
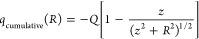
4Figure 3 reports the cumulative charge distributions for the C_60000_ nanoflake interacting with a negative unit charge at (0,0,5) and (0,0,10) a_0_, Figures 3a and 3b, respectively, as described by the MMÅ2 model with net induced charge constrained to be either +1 or 0. The corresponding charge distributions given in eq 4 are also reported. To obtain the results for the MMÅ2 model, we sum the charges of all C atoms within radius *R*.

**Figure 3:**
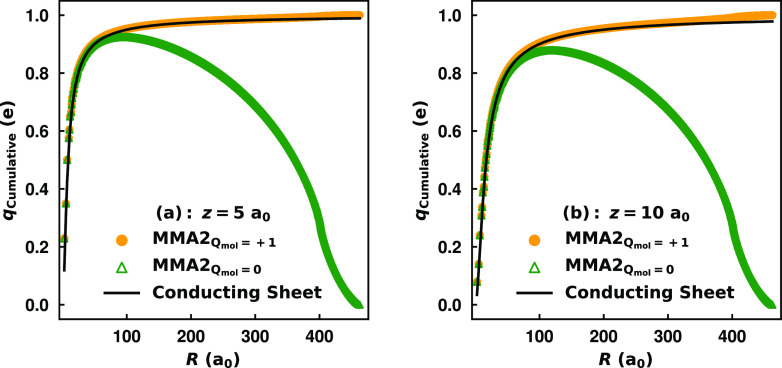
Cumulative charge of a C_60000_ nanoflake induced by a point charge (*Q* = −1) at (0, 0, *z*) as described by the charge flow contribution of the MMÅ2 model when the nanoflake is grounded (*Q*_mol_ = +1) or ungrounded (*Q*_mol_ = 0). The cumulative charge for a thin conducting slab is included for comparison. The cumulative charge is plotted with respect to the radial distance from the center of the carbon sheet. Two cases are considered: (a) the negative point charge is 5 and (b) 10 a_0_ above the molecular plane along the principal rotation axis.

Overall, there is good agreement between the charge distributions from the FQ model (with a net molecular charge of +1) and those from the classical treatment of a grounded metallic sheet. When the net induced charge is constrained to be zero, the charge distribution of C_60000_ induced by the point charge and calculated using the MMÅ2 model closely follows that of eq 4 up to about R = 80 a_0_, after which the induced charge switches sign. It is important to note that the change of sign in the induced charge for large R values proves relatively unimportant to the electrostatic potential above (or below) the middle of a nanoflake with 60,000 atoms provided *z* is much smaller than the molecular radius. This is because the interactions between the external charge and induced atomic charges in the short to intermediate range dominate the electrostatics near the central ring when *z* ≪ *R*.

The results presented above demonstrate that the overestimation of the long-range charge transfer is not a major problem when the MMÅ2 model is applied to large hexagonal PAHs or carbon nanoflakes. We believe that there are two factors that act to prevent the breakdown of the model:

1. The increasing metallic behavior as one progresses to larger systems.
2. The repulsive interactions between the induced charges on C atoms near the periphery of the molecule.

It is well-known that an improved description of the polarization potential resulting from a point charge interacting with a conducting sheet includes a shift in the “image plane” which accounts for the displacement of the charge distribution outside the plane of atoms.^46−48^ This image plane shift results in a −*Q*^2^/(4|*z* – sgn(*z*) × *z*_im_|) potential, where *z*_im_ gives the location of the image plane, and sgn(*z*) is included to reflect the fact that the systems of interest are isolated and are symmetric with respect to reflection in the *xy* plane. In a recent study a value of *z*_im_ = 1.98 a_0_ was deduced for the image plane of graphene.^48^ Obviously, an FQ model allowing only in-plane charge redistribution cannot account for the image plane shift. However, the full MMÅ2 model, including both point inducible dipoles and charge flow terms, can partially account for this effect. This can be seen in Figure 4 which compares the polarization potential for C_60000_ from the full MMÅ2 model to −1/(4|*z* – 1.98|).

**Figure 4:**
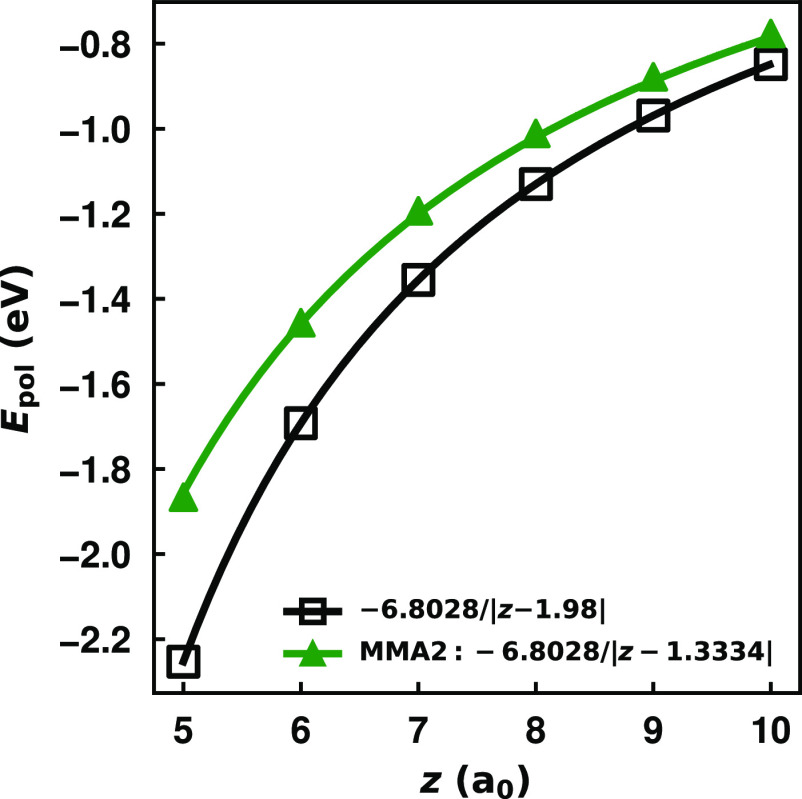
Polarization potential due to a negative point charge (*Q* = −1) interacting with a grounded (*Q*_mol_ = +1) C_60000_ nanoflake, as described by the full MMÅ2 model with both charge flow and inducible dipoles. The shifted-plane classical image potential is included for reference with the image plane parameter set to *z*_im_ = 1.98 a_0_, as determined in ref (48). A fit of the polarization potential from the MMÅ2 model to a function of the form −6.8028/|*z* – *b*|, where *b* is a free parameter, is reported.

Overall, there is fairly good agreement between the two potentials, although that from the MMÅ2 model is less attractive than the shifted image plane result, with the difference being as large as 16% at *z* = 5 a_0_. While this could reflect a deficiency in the inducible atomic dipoles of the MMÅ2 model, we note that if *z*_im_ is reduced to 1.3334 a_0_, the polarization potential from the MMÅ2 model and the shifted-plane image potential would be in excellent agreement. This can be seen in the curve fit to the MMÅ2 data in Figure 4. Values of *z*_im_ as small as 1 a_0_ have been considered in the literature.^49^

There has been considerable debate in the literature^50−55^ as to whether a single layer of graphene is fully screening, i.e., whether a graphene sheet fully screens out the interactions of molecules on one side of a graphene surface from molecules or a solid substrate on the other side of the sheet. Much of this debate has centered around whether a substrate, in the absence of charge transfer between the substrate and the graphene layer, impacts the contact angle of a water droplet on the other surface. If graphene were realistically described as a thin conducting slab, then the net interaction energy of two point charges (*Q*) at distances *z* and −*z* from the sheet would be −*Q*^2^/(2|*z*|), independent of whether the charges are of the same or of a different sign. This is simply the sum in the interactions of the point charge on each side with its induced charge distribution.

In Figure 5 we report the net energy of the C_60000_ system with two point charges of −1 on opposite sides of the sheet (at *z* and – *z*) as described by the MMÅ2 model, retaining only the charge flow terms. Note that the net charge of the nanoflake is constrained to be +2.

**Figure 5:**
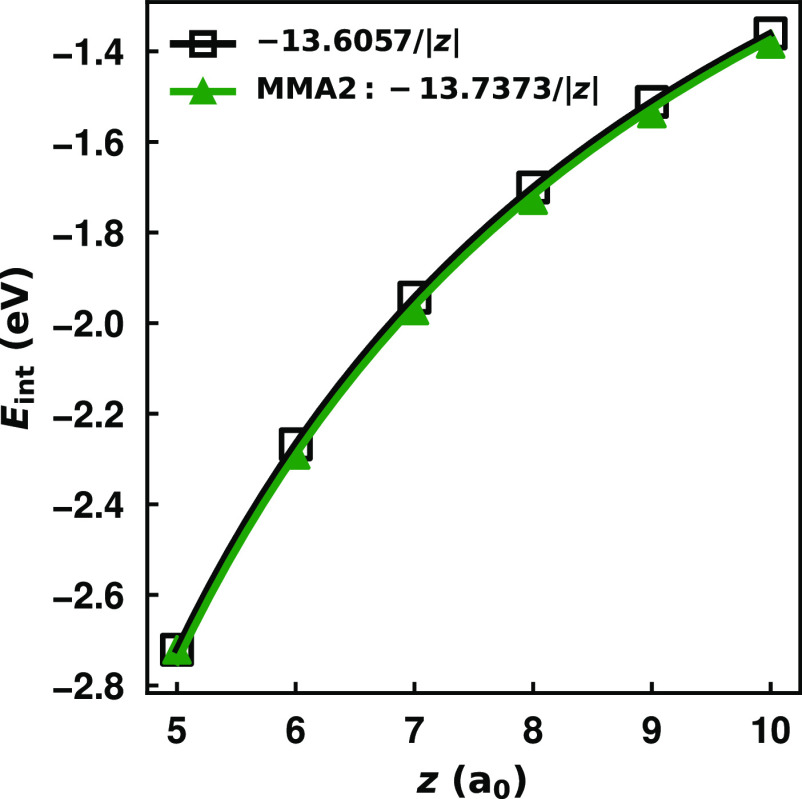
Total interaction of two negative point charges on opposing sides of a grounded (*Q*_mol_ = +2) C_60000_ nanoflake as described via the charge flow contribution of the MMÅ2 model. The classical image potential solution for an intervening conducting sheet, −1/(2|*z*|) (a.u.), is included for reference. A fit of the model potential to a function of the form *A*/|*z*|, where *A* is a free parameter, is included.

The net interaction energy for this system obtained using the charge flow component of the MMÅ2 model is nearly identical to that of −1/(2|*z*|), the classical image potential result. However, in the MMÅ2 model the two point charges induce a cumulative charge redistribution in the plane of the atomic sites in contrast to the classical result for a conducting sheet, where each point charge induces a separate charge redistribution in the surface it faces. If the two point charges are of opposite sign, then the net interaction energy for a conducting sheet is still −1/(2|*z*|). Although the MMÅ2 model also reproduces this result, the “physics” is quite different. In the charge flow contribution of the MMÅ2 model, there is no induced charge when the two point charges are of opposite sign, and one simply has the interaction between the two point charges. In the conducting slab model, the point charge on each side of the sheet induces a charge distribution of opposite sign on the side that it faces. These external charge-induced charge distribution interactions screen the direct interaction between the two external point charges. While one might conclude that the MMÅ2 model is getting the “right answer for the wrong reason” we believe that it actually provides a more realistic description of charge flow polarization in graphene and in large hexagonal carbon nanoflakes than does the conducting slab model. In other words, we believe that an atomically thin molecule or extended system does not fully screen interactions between charges on its opposite faces.

Although not shown in this article, we also calculated for a C_15000_ nanoflake the polarization energy resulting from the interaction of two point charges of like and opposite sign when point inducible charges and dipoles are included in the MMÅ2 model. When the external charges are of opposite sign (±1), the polarization of the nanoflake energy is entirely due to the *z*-component of the inducible dipoles. This energetic contribution is smaller than the Coulomb attraction between the external point charges and is approximately proportional to *z*^–2^. In contrast, when the two point charges are of the same sign, the contribution from the inducible dipoles originates from the in-plane components (*x* and *y*) only. This energetic contribution is small relative to both the Coulomb repulsion between the external charges and the attraction between the external and induced charges.

In this work, we have shown that a modified Mayer-Åstrand model (denoted MMÅ2) including both point inducible dipoles and fluctuating charge polarization terms is able to accurately describe the dipole polarizabilities of small PAHs and, for sufficiently large graphene nanoflakes, reproduces the classical image potential for a conducting sheet. To the best of our knowledge, this is the first application of coupled fluctuating charges and inducible dipoles in modeling the behavior of large carbon nanoflakes. We have demonstrated that the polarization behavior of extended systems, such as graphene, can be modeled via large finite analogues. The MMÅ2 model is expected to be well suited to describing polarization interactions in the adsorption of ions and molecules on large PAHs, graphene nanoflakes, and graphene itself. In the near future, we plan on applying this polarization model to study the nonvalence anionic states of these finite nanoflake systems with the hope of demonstrating their connection to the image potential bound states of graphene.

## Data Availability

The data underlying this study are openly available in the repository CE_polarization_of_large_nanoflakes at https://github.com/dev-m-mulvey/CE_polarization_of_large_nanoflakes.git.
